# The Impact of Expatriates’ Cross-Cultural Adjustment on Work Stress and Job Involvement in the High-Tech Industry

**DOI:** 10.3389/fpsyg.2019.02228

**Published:** 2019-10-09

**Authors:** Min Chen

**Affiliations:** Academy of Financial Research, Wenzhou University, Wenzhou, China

**Keywords:** expatriate, cross-cultural adjustment, work stress, job involvement, personal psychological adjustment

## Abstract

The personal traits of expatriates influence their work performance in a subsidiary. Nevertheless, organizations tend to hire candidates who are suitable from the technological dimension but ignore personal and family factors. Expatriates might not be familiar with a foreign place, and most organizations do not provide the so-called cultural adjustment training. The selected expatriates often accept the job without knowing the future prospects of their career, which can result in individual and family turmoil initially. Moreover, the unknown future career prospects and concern over when they will return to the parent company can affect expatriates’ work. Cross-cultural competence refers to the ability of individuals to work effectively and live normally in different cultural contexts, and this ability requires expatriate employees to adopt adaptive thinking patterns and behaviors in the host country. To explore the effect of expatriates’ cross-culture adjustment on their work stress and job involvement, this study therefore uses an empirical approach in which data are collected with a questionnaire survey and proposes specific suggestions, according to the results, to aid expatriates in their personal psychological adjustment. The results show that the challenges faced by expatriate employees are derived from assigned tasks, unknown environments, language barriers, and cultural differences. Excessive pressure will impose ideological and psychological burdens upon the expatriates and even lead to physical symptoms, however, the appropriate amount of pressure can play a driving role and promote the smooth progress of the work. High-tech industry employees who can adapt to the customs and cultures of foreign countries have higher work participation and are more likely to find ways to alleviate work stress. It has also been found that the stronger the cross-cultural competence of employees, the better their adjustment to the host country and the higher their corresponding job performance.

## Introduction

When developing countries began to attract foreign businesses with land and tax incentives, many foreign-capital enterprises stationed in developing countries. In particular, the high-tech industry invested the most in developing countries. In the initial period of openness, small and medium enterprises appeared most often; later, large enterprises gradually moved in. In consideration of technology transfer and the establishment of corporate management systems for offshore sourcing, as well as the demands for market expansion, numerous expatriates are required for executing company tasks in subsidiary companies from the initial establishment and planning, factory founding, marketing, technology, and general affairs. Such expatriates’ personal traits and work performance are correlated with the success of subsidiary companies. Once these employees are incorporated into production activities, many manufacturing supervisors for expatriation management and technology transfer are required. For this reason, expatriates with technologically suitable work experience are first considered by an organization, while the problems of personal and family factors are relatively ignored. Such expatriates might not be familiar with a foreign place, and most organizations do not provide relevant cultural adjustment training before the expatriation, while the selected expatriates, based on the desire to serve the company, will accept the offer without knowing their career prospects. In this case, accepting the assignment does not necessarily mean that the employees are eager to accept. Leaving for an overseas location without personal willingness can cause an expatriate to feel the loss of future career prospects and worry about the return to the parent company after the term of employment overseas. Relevant measures taken by an organization to rotate expatriates according to a known timeline and more clearly defining the expatriates’ career trajectory in the company would reduce the hesitation of expatriates. Cross-cultural adjustment problems caused by various differences in life and social culture also influence an expatriate’s work stress. With an increasing number of expatriates, expatriation expenses are rather high for an enterprise and individuals. International assignments that do not meet the target not only incur economic costs to multinational corporations but also damage future cooperation with subsidiaries; moreover, expatriates may lose their self-esteem and have negative attitudes, which may result in resignation. Expatriate management is no longer a fringe topic in human resource management in China but a serious challenge that is urgent in both the practical and academic circles. An enterprise therefore should emphasize the success of expatriation and provide the necessary support for the expatriate’s career. In addition to real subsidies, a well-defined process is necessary for the selection, training, overseas life, overseas work, and return. Furthermore, assistance in emergencies and the relief of expatriates’ anxiety are essential. In certain developed countries, research on expatriate management started as early as the 1970s, however, in China, this research has begun more recently. Thus, a systematic system of research on expatriate management has not yet been formed. The current theories and research are primarily collations and analysis of foreign literature. In summary, there are still vacancies in this research field. From the perspective of changing the work pressure and job performance of foreigners after cross-cultural adjustment, case studies are conducted through questionnaires to analyze the impacts of assignments on employees, providing reasonable adjustment advice for enterprises and employees themselves in the case of expatriates.

## Literature Review

### Cross-Cultural Adjustment

Rui and Wang (2015) defined cross-cultural adjustment as culture shock generated in the process of an individual adjusting to a different culture in a foreign country. Collie et al. (2015) regarded cross-cultural adjustment as individual psychological stress relief, when encountering cross-culture shock in different environments, to reduce conflict and stress at work or outside of work and achieve psychological comfort and ease (Stilianos et al., 2017). Abdullah et al. (2015) revealed that from one specific culture to another culture, an individual had to readjust to cultural differences and change the accustomed lifestyles and thinking principles; in the cross-cultural experience, an individual would acquire distinct perception changes and physical and mental changes. This process was referred to as cross-cultural adjustment. Krishnan and Kirubamoorthy (2017) regarded cross-cultural adjustment as the interaction among people with a distinct culture. Such interaction behaviors were communicated through languages.

According to Chen et al. (2018), culture includes art, sports, cooking, music, dance, architecture, history, and family. The expression of culture is different in various countries, and successful enculturation of expatriates includes four dimensions.

**Self-adjustment:** Self-adjustment mainly reinforces the well-being of an expatriate who feels that they are being respected and trusted. An expatriate with better adjustment can more easily deal with contradiction, emotional depression, and loneliness among people.

**Other adjustments:** This is an essential factor in developing a permanent and stable friendship with local people and being glad to communicate with local people for better enculturation.

**Cognitive feeling:** This factor refers to reducing uncertainty in interpersonal relationships, decreasing suspicions among people, and avoiding misunderstandings caused by cultural differences.

**Cultural toughness:** Different countries present distinct cultural characteristics. Expatriates of transnational enterprises in some countries show different forms of dissatisfaction, including job dissatisfaction, stress, medical insurance, entertainment, food, and working skills of colleagues.

### Work Stress

Li and Zizzi (2017) asserted that work stress was a unique reaction to the interaction between the attitudes toward supervisors and the frequent and strong workplace-related conflicts. Bijwaard and Wang (2016) reported that work stress has a bad physiological and psychological influence on a worker in an organization (or an institution) when individual capability cannot live up to the corresponding expectation. Milbourne and Wilkinson (2015) defined work stress as the spiritual fatigue caused by being slowly exposed to heavy work stress; when the person felt exhausted and emptied, such physical and mental exhaustion at work would result in the lack of work enthusiasm, high frustration, nervousness, and even insomnia, headache, anxiety, and depression (Song et al., 2015). Gullo et al. (2015) divided stressors into interpersonal relationship stressors, task relationship stressors, organizational relationship stressors, and physical and mental relationship stressors.

According to Yu et al. (2018), four stress measurements for expatriates are applied in the present study.

**Interpersonal relationship:** Interpersonal relationship stressors include local people’s characters, local people’s work characters, the poor relationship among work teams, and work–family conflict.

**Task relationship:** Task relationship stressors include large workload, unclear tasks, and task conflicts.

**Organizational relationship:** Organizational stressors are related to the organizational pattern, management model, and organizational support.

**Physical relationship:** Physical and mental relationship stressors include daily life, inconvenient transportation, and unfair treatment.

### Job Involvement

Job involvement refers to the identification of an individual psychologically at work, which is also a meaningful index of working attitude (Honebein and Honebein, 2015). The job is the focus of an individual, and job involvement refers to the work attitudes and satisfaction with current work. Job involvement can be divided into two dimensions, namely, the degree to which an individual is involved in specific work and the enthusiastic participation and individual initiative to improve one’s work in comparison with other work (Rujipak and Limprasert, 2016). Ciocca et al. (2015) regarded job involvement as a worker’s willingness and acceptance of current work. Reddington et al. (2015) defined it as the performance of work tasks and divided job involvement into individual and group dimensions. Shen and Jiang (2015) stated that job involvement was individual self-dignity related to one’s identity with the work content and work performance. For a group, job involvement reflects organizational commitment.

Referring to Li (2018), the psychological conditions of job involvement are classified into three types:

**Meaningfulness:** Meaningfulness is defined as being rewarded for one’s work role. It is generally considered that the most important source of deriving meaning from work is to receive feedback after engaging in work activities.

**Safety:** Safety refers to working conditions under which negative effects on employees’ well-being. Each industry causes certain effects on society. For this reason, it is important for employees to be comfortable with job involvement.

**Availability:** Availability is defined as an employee perceiving actual, psychological, or emotional assistance at work.

### Correlations Between Cross-Cultural Adjustment and Work Stress

Chen et al. (2018) noted the physical and spiritual stress during expatriation and proposed that individual handling methods should include planning solutions and re-evaluating problem-solving tools (Stilianos et al., 2017), while emotional handling should include stress release, avoiding efforts to escape from reality, and seeking social assistance. Jyoti and Kour (2015) indicated that an expatriate should be able to effectively navigate the cross-cultural environment, including through the maintenance of psychological health and well-being, as well as self-confidence and effective stress management. At the beginning of expatriation, an individual may be anxious about the strange environment. Proper local guidance and necessary directions for work offered before the expatriation can be of great help for an expatriate (Song et al., 2015).

### Correlations Between Work Stress and Job Involvement

Yao et al. (2015) revealed that most work stressors of a firefighter, with the exception of external organization (public opinions), were positive, reflecting that the formation of internal or individual work stress can have positive effects on job involvement. Sorrells (2015) concluded that higher work stress was correlated with lower job involvement. Li and Zizzi (2017) found contradictory tasks, blurred tasks, and heavy tasks to be the major factors in work stress, resulting in spiritual fatigue, loss of one’s identity, and depression of mood. Work stress was correlated with a heavy workload and task contradictions, and heavy workload was shown to reduce job involvement (Honebein and Honebein, 2015).

### Correlations Between Cross-Cultural Adjustment and Job Involvement

Yu et al. (2018) suggested that an individual should first adjust to life overseas adjustment when abroad, as an individual’s mood at work can be negatively affected if they have not adjusted to life outside of work (Rujipak and Limprasert, 2016). In research on the influence of overseas adjustment on performance in accordance with company expectations, the ability to work overseas until the end of the contract, the establishment of normal social relationships overseas, and successfully coping with stress, Li (2018) discovered that overseas adjustment would affect job involvement. Sharma and Juyal (2017) also showed that foreign workers with higher adjustment to the host country would present higher job involvement. Consequently, an employee with higher adjustment showed better performance at work (Shen and Jiang, 2015).

Based on previous research ideas and results, the following research hypotheses are proposed in this study:

(1) Cross-cultural adjustment has a significant negative impact on work pressures of employees.
(2) Work pressure has a significant negative impact on job involvement.
(3) Cross-cultural adjustment has a significant positive impact on job participation.
(4) Cross-cultural adjustment has a significant negative impact on job performance.

To obtain detailed information, a questionnaire was used for data collection. The questions used in the questionnaire were extracted from well-established foreign questionnaires (see Appendix). First, the standard translation was carried out; then, the translated content was modified and integrated, and after screening and sorting, a scientific and complete questionnaire was prepared. In addition, in the choice of questions, the relevant research of domestic scholars also played an important reference role. To enable the respondents to clearly specify their choices and evaluations in response to the reference questionnaire, the questionnaire employed a 5-point Likert scale, which makes the final survey results more accurate.

### Selection of Control Variables

One-way ANOVA was utilized to study and analyze demographic variables. Significant differences were found to exist between the years of work and cross-cultural competency, while no significant difference was found between the variables of job performance; other demographic variables exhibited no significant differences on the basis of two research variables. Therefore, in the subsequent analysis and tests, the working years were selected as the control variables for research.

### Variable Measurement Scales

Since the research results of cross-cultural competence carried out by Koester and Olebe (1988) were widely accepted and influenced the academic community, in this study, the results of their research were used to conduct a survey on cross-cultural competency. The specific content can be divided into abilities such as cross-cultural communication, professional knowledge, professional skills, and interpersonal skills. The specific measurement entries for cross-cultural competence are shown in Table 1.

**TABLE 1:** Measurement entries of cross-cultural competence.

CC01	I like to communicate with people from different cultural backgrounds.
CC02	I can properly resolve conflicts with people from different cultural backgrounds.
CC03	When people from different cultural backgrounds need help, I will make suggestions in a way that they can understand.
CC04	In different cultural backgrounds, I can tolerate higher uncertainty.
CC05	In different cultures, I refuse to do what I don’t want to do.
CC06	Under different cultural backgrounds, I will take the initiative to introduce myself to the people I want to know.
CC07	When communicating with people from different cultural backgrounds, I am very confident.
CC08	When communicating with people from different cultural backgrounds, I will reflect and provide feedback on the conversation.
CC09	When communicating with people from different cultural backgrounds, I will change my position.
CC10	When communicating with people from different cultural backgrounds, I can express my views clearly.
CC11	When communicating with people from different cultural backgrounds, I will actively express my ideas.

Job performance includes task performance and contextual performance, which are separately measured. The task performance can be reflected in the aspects of task quantity, task quality, and completion efficiency, while the contextual performance can be measured by the research results of scholars (Van Scotter et al., 2000). The scale has high validity and reliability. The specific entries for job performance measurement are shown in Table 2.

**TABLE 2:** Measurement entries of job performance.

TP01	I have the expertise to accomplish the task.
TP02	I have the ability to accomplish tasks.
TP03	I show good judgment in accomplishing tasks.
TP 04	I can accomplish the task accurately.
TP05	I show creativity in accomplishing tasks.
TP06	I uphold the highest professional standards.
CP01	I encourage others to overcome interpersonal barriers and get along well.
CP02	At work, I can be self-disciplined and self-restrained.
CP03	I am very concerned about the important details of my work.
CP04	To complete the task on time, I will use break time to work.
CP05	I will seek the cooperation of colleagues and relevant departments to complete the work.
CP06	I often take the initiative to solve difficult work enthusiastically.
CP07	I take the initiative to solve problems in my work.
CP08	I will overcome difficulties to complete the task.

### Reliability and Validity Tests

The entries of the questionnaires in this study are based on the research results of domestic and international researchers; consequently, the questionnaires have shown certain content validity. Measurements of cross-cultural adjustment, work stress, and job involvement are tested for overall structural causation, and the analysis results of the linear structural relation model (LSR model) reveal the overall fitness of the LSR model, which shows good convergence and predictive validity. The item-to-total correlation coefficients are used to determine the construct validity of the questionnaires, i.e., the reliability test. The item-to-total correlation coefficients are calculated and applied to evaluate the content of questionnaires. In this study, the item-to-total correlation coefficients of the measurements were higher than 0.7, revealing a certain degree of measurement construct validity.

To further verify the reliability of the questionnaire, reliability analysis was performed. The formal questionnaire was developed according to the standard, and the measured Cronbach’s α was in the range of 0.70 to −0.90, which is in conformity with the range of reliability.

### Data Collection

Based on the negotiations with the relevant persons responsible for given enterprises, the questionnaires were mainly issued and circulated by the enterprises in a direct manner. In response to the high-tech industry, 500 questionnaires were distributed to foreigners in high-tech industries in Zhejiang Province, and 410 questionnaires were collected. The response rate of all questionnaires was 82%. To ensure that each questionnaire could provide valid survey results, in the process of collecting the questionnaires, the invalid questionnaires were treated as “obsolete;” 33 questionnaires were discarded, while 377 valid questionnaires were finally collected. The screening of invalid questionnaires was carried out in strict accordance with the standards. The criteria adopted were as follows: First, the questionnaires were not carefully filled out; for example, the same answer was selected for all questions in the questionnaire or two answers were alternately selected. Second, the questions in the questionnaires were incomplete, with many short answers and blanks. The final response rate of valid questionnaires was 75.4%, which met the requirements for validity.

## Analysis of Empirical Results

### Establishment of the SEM Model and Application of LISREL

**FIGURE 1:**
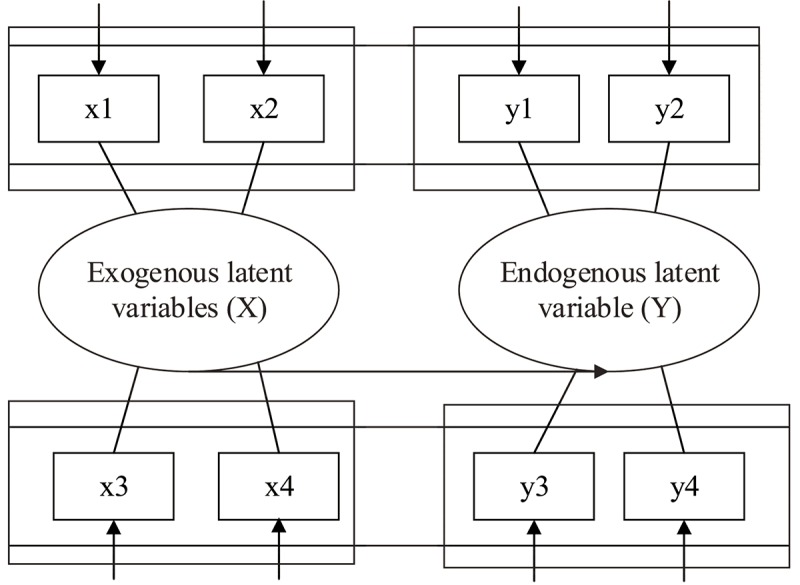
The structure of the structural equation model (SEM).

**FIGURE 2:**
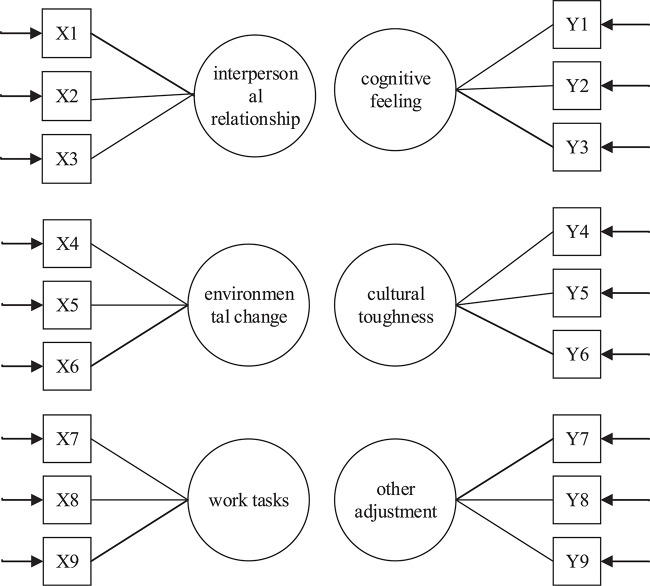
The pressure sources of expatriates based on linear structural relation (LISREL) model.

Many of the variables involved in social and psychological research are often not accurately and directly measured, and these variables can be used as latent variables, such as work stress and job satisfaction. Therefore, these latent variables can only be measured with observable indicators, such as the detection indicators of work autonomy (latent variable), of work mode selection and work target adjustment, as well as indicators of job satisfaction such as work interest, work pleasure, and work preference (observable indicators). When exploring the relation between external work pressure and the factors arising from external and internal sources, the traditional linear regression model can only deal with the problem with one dependent variable; it cannot measure and deal with subjectively strong variables. There is a certain relation between the variables, and this relation can be calculated. The calculated value is called the parameter. The size of the parameter value indicates the impact of the indicator on the pressure of the employee. Therefore, the parameter can guide the enterprise to achieve the purpose of quickly improving the personal and business efficiency of employees. Therefore, in this study, the work pressure of expatriate employees was analyzed and discussed by constructing a structural equation model (SEM). The structure of the SEM model is shown in Figure 1. The design of the pressure source of the expatriate employee based on the linear structural relation (LISREL) model is shown in Figure 2.

### Evaluation Indicator of the LISREL Model

The LISREL model, combining factor and path analysis in traditional statistics with a set of equations including two or more variables for which there are values that can satisfy all the equations simultaneously in econometrics, can calculate multiple factors and causal paths at the same time. The fitness of the model can be evaluated with primary fitness criteria, including the overall model fitness and internal structural fitness of the model. The data are classified as below, and the primary fitness, internal fitness, and overall fitness of the model are demonstrated as follows.

Table 3 reveals that four measurements of cross-cultural adjustment (self-adjustment, other adjustments, cognitive feeling, cultural toughness) significantly influence cross-cultural adjustment (*t* > 1.96,*p* < 0.05), four measurements of work stress (interpersonal relationships, task relationship, organizational relationship, physical relationship) present significant effects on work stress (*t* > 1.96,*p* < 0.05), and three measurements of job involvement (meaningfulness, safety, availability) significantly affect job involvement (*t* > 1.96,*p* < 0.05). Apparently, the primary fitness of the overall model is good.

**TABLE 3:** Analysis results of the overall linear structural relation (LSR) model.

**Evaluation item**	**Parameters/standards**		**Results**	***T* values**
Primary fitness	Cross-cultural adjustment	Self-adjustment	0.702	9.45^∗∗^
		Other adjustment	0.713	10.12^∗∗^
		Cognitive feeling	0.723	10.36^∗∗^
		Cultural toughness	0.731	10.97^∗∗^
	Work stress	Interpersonal relationship	0.784	14.15^∗∗^
		Task relationship	0.775	13.44^∗∗^
		Organizational relationship	0.766	12.69^∗∗^
		Physical relationship	0.754	12.33^∗∗^
	Job involvement	Meaningfulness	0.806	16.27^∗∗^
		Safety	0.811	17.38^∗∗^
		Availability	0.791	15.82^∗∗^

*^∗∗^*p* < 0.01.*

Table 4 shows positive and significant correlations between cross-cultural adjustment and work stress (−0.873, *p* < 0.01), work stress and job involvement (−0.862, *p* < 0.01), as well as cross-cultural adjustment and job involvement (0.884, *p* < 0.01), which respectively, support the first three hypotheses.

**TABLE 4:** Analysis results of overall linear structural relation (LSR) model.

**Evaluation items**	**Parameters/standards**	**Results**	***T* values**
Internal fitness	Cross-cultural adjustment → work stress	−0.873	−24.86^∗∗^
	Work stress → job involvement	−0.862	−22.57^∗∗^
	Cross-cultural adjustment → job involvement	0.884	27.51^∗∗^

*^∗∗^*p* < 0.01.*

In Table 5, the overall fitness of the LSR model is χ^2^/*Ddf* = 1.476, which is less than the standard value 3. In addition, the value of the root mean square residual (RMR) is 0.004, which shows that the values of χ^2^/*DFdf* and RMR are acceptable. Furthermore, the chi-square test is sensitive to sample sizes; therefore, it is not applicable to the direct determination of fitness. The overall fitness standards of the LSR model are goodness of fit index (*GFI*) = 0.968 and adjusted GFI (*AGFI*) = 0.915, which are higher than the standard value of 0.9 (the closer the GFI and AGFI are to 1, the better the model fitness). Therefore, better fitness indexes are proposed.

**TABLE 5:** Analysis results of the overall fitness of the linear structural relation (LSR) model.

Overall fitness	χ^2^/*Ddf*	1.476
	GFI	0.968
	AGFI	0.915
	RMR	0.004

## Conclusion

During their first experience with expatriation, employees often encounter unexpected situations, which are often accompanied by cultural setbacks and the risks of failure to complete assignments, while increasing the anxiety and distrust of expatriate employees. Whether an expatriate employee can successfully navigate the setback period and overcome the anxiety and dissatisfaction will directly affect their adaptation in the cross-cultural environment, thus affecting their job performance in the host country. Individuals who have strong cross-cultural competence can take advantage of relevant knowledge and skills to promote the smooth development of cross-cultural employment. Improving their acceptance of an unfamiliar culture and integrating it with the cultural environment can enable expatriate employees to fully adapt to the new environment and get along with relevant personnel; the ability to recognize the differences between the old and new environment as soon as possible can help the expatriate process the possible conflicts to achieve the goal of integration with the cross-cultural environment and meet the requirements for high job performance.

In this study, the control variables of respondents were collected, such as gender, age, working years, and jobs. However, in data processing, in addition to the influence of working years on cross-cultural competence, other variables are associated with independent variables. There are no significant relations between dependent variables, control variables, and independent variables. Cross-cultural competence is positively related to the performance of expatriate employees. The stronger the cross-cultural competence of an expatriate employee is, the better the performance in the host country is and the higher the corresponding job performance is. The research results have shown that the ability of expatriate employees of high-tech industries to adjust to the customs in the host country improves their job involvement. This effect might occur because employees in high-tech industries who are able to adjust to life in the host country can more easily find ways to release work stress, e.g., shopping or travel. This skill would help employees in high-tech industries reduce job stress and achieve greater involvement in the job. Generally, local people, expatriates, and local employees all have stress-releasing methods after work. Expatriates in the high-tech industry who feel enjoyment in life experience reduced work stress and perform better on the job. Expatriates’ cross-cultural adjustment is therefore correlated with work stress and job involvement. Expatriates in the high-tech industry who are able to easily control their work appear to experience lower work stress and more easily exhibit job involvement. In general, expatriates in the high-tech industry who are able to adjust to their work contexts feel less frustration and work stress, allowing them to reach higher achievement and higher job involvement.

The success of expatriates is a classic topic in international practices and academic research on human resource management. Pressures of expatriate tasks on employees can be positive and negative. There are many studies on negative factors, such as cultural differences, lifestyle changes, language communication barriers, and other factors that will impose psychological burdens upon employees. If the negative pressures are accumulated instead of relieved, the expatriate’s job performance may be influenced. However, positive pressures can play a dynamic role and can support expatriates to take multi-objective initiatives to pursue individual success at the individual, work, and organizational levels.

## Recommendations

Based on the findings analyzed in this study, the following practical suggestions and recommendations are proposed:

Managers in the high-tech industry should consider the impact of expatriation period on employees; a longer expatriation period will result in higher stress. Expatriates are susceptible to a distinct source of stress, and strong organizational support could help them adjust to local life and work conditions and enhance job efficiency.

Expatriates’ work stress is closely related to their ability to adjust to local conditions. Although the climate cannot be changed, a high-tech business could satisfy expatriates’ expectations for a living environment as much as possible to facilitate good interactions with local people.

An expatriate who does not adjust to the expatriate life will not present high job involvement. In this case, a high-tech business could express concern about the expatriate’s daily life and provide activities to help the expatriate understand the customs of the host country and become further accustomed and integrated into local life to enhance his or her job involvement.

The performance of expatriate employees is influenced by cross-cultural competence. Therefore, enterprises can establish an effective expatriate selection system. According to the actual situations of the host countries, the elements of individual cross-cultural competence related to the expatriate’s tasks can be evaluated and cross-cultural competence and individual adaptability can be effectively combined, allowing potential expatriates to be matched with appropriate host countries to obtain the optimal fit.

## Summary and Future Research

In this study, the work pressures of expatriate employees are comprehensively understood based on questionnaires administered to foreign employees in high-tech industries, the sources and impacts of stressors are analyzed, and recommendations for the allocation of human resources for enterprises and individuals are presented to promote the common development of individuals and enterprises.

The research objects of this study are the staff of the high-tech industry, most of whom are technical talents; therefore, the requirements for interpersonal communication are relatively low at work. For certain positions that require frequent communication with customers and supervisors, the expatriate’s tasks will be more versatile in terms of different cultural backgrounds and language atmospheres of employees; thus, further research is needed in this field.

## Data Availability Statement

The raw data supporting the conclusions of this manuscript will be made available by the authors, without undue reservation, to any qualified researcher.

## Ethics Statement

Ethical review and approval was not required for the study on human participants in accordance with the local legislation and institutional requirements. The patients/participants provided their written informed consent to participate in this study.

## Author Contributions

The author did the literature search, figures, study design, data collection, data analysis, data interpretation, writing etc.

## Conflict of Interest

The author declares that the research was conducted in the absence of any commercial or financial relationships that could be construed as a potential conflict of interest.

## Appendix

### Interview and Survey on the Work and Life Adaptability of Foreigners in High-Tech Industry in Zhejiang Province

1. Life changes
  (1) When did you come to work in Zhejiang?
  (2) How do you feel about Zhejiang as a whole?
  (3) Does the new environment affect your old habits?
  (4) Is the work in Zhejiang similar to what you imagined?
  (5) How will your new job affect you?
2. Adaptation time
  (1) How long do you think it will take to integrate into the environment here?
  (2) Have you communicated with your neighbors?
  (3) How long did it take you to adjust your working state?
  (4) Do you think you need to continue to adapt to the existing environment?
  (5) Does the company help you adapt to the new environment?
3. Social support
  (1) Does your family support you to work in Zhejiang?
  (2) Are there any colleagues assigned to work in Zhejiang?
  (3) Does the company provide you with relevant adaptation support?
  (4) Have you ever received help from others in your life or work?
  (5) If you have any difficulties, who will you turn to for help?
4. Impression of Chinese culture
  (1) Does the company systematically introduce Chinese culture to you?
  (2) What do you think of the Chinese culture with Zhejiang regional characteristics?
  (3) Do you feel Chinese culture in your work?
  (4) Do you feel that you are being influenced by Chinese culture?
  (5) Will you share Chinese culture with your friends?
5. Cognitive style
  (1) What is your impression of high-tech jobs?
  (2) How do you feel when you see the way you work in reality?
  (3) Have you ever encountered any inconvenience in your company’s life?
  (4) What do you think of the cost of living in Zhejiang?
  (5) Has the company arranged accommodations for you?
6. Personality factors
  (1) Do you feel pressure during your work?
  (2) Where do you think the pressure comes from?
  (3) Do you think that such pressure can be converted into power?
  (4) How do you deal with the above pressure?
  (5) Does the company provide you with psychological counseling support?
7. Knowledge and skills
  (1) Do you know Chinese?
  (2) Did you study Chinese before you came to work in the country?
  (3) Do you think Chinese is helpful for your work?
  (4) Can you communicate with other employees in Chinese?
  (5) Does the company provide Chinese training support for you?

### Cross-Cultural Competence Survey

1 = strongly disagree, 2 = slightly disagree, 3 = uncertain, 4 = slightly agree, 5 = strongly agree.

**Table:**

**Serial number**	**Problem**	**1**	**2**	**3**	**4**	**5**
1	I like to communicate with people from different cultural backgrounds.					
2	I can properly resolve conflicts with people from different cultural backgrounds.					
3	When people from different cultural backgrounds need help, I will make suggestions in a way that they can accept.					
4	In different cultural backgrounds, I can tolerate higher uncertainty.					
5	In different cultures, I refuse to do what I don’t want to do.					
6	Under different cultural backgrounds, I will take the initiative to introduce myself to the people I want to know.					
7	When communicating with people from different cultural backgrounds, I am very confident.					
8	When communicating with people from different cultural backgrounds, I will reflect and feedback on the conversation.					
9	When communicating with people from different cultural backgrounds, I am open to changing my position.					
10	When communicating with people from different cultural backgrounds, I can express my views clearly.					
11	When communicating with people from different cultural backgrounds, I will actively express my ideas.					

### Job Performance Survey

1 = strongly disagree, 2 = slightly disagree, 3 = uncertain, 4 = slightly agree, 5 = strongly agree.

**Table:**

**Serial number**	**Problem**	**1**	**2**	**3**	**4**	**5**
1	I have the expertise to accomplish the task.					
2	I have the ability to accomplish tasks.					
3	I show good judgment in accomplishing tasks.					
4	I can accomplish the task accurately.					
5	I show creativity in accomplishing tasks.					
6	I uphold the highest professional standards.					
7	I encourage others to overcome interpersonal barriers and get along well.					
8	At work, I can be self-disciplined and self-restrained.					
9	I am very concerned about the important details of my work.					
10	To complete the task on time, I will use break time to work.					
11	I will seek the cooperation of colleagues and relevant departments to complete the work.					
12	I often take the initiative to solve difficult work enthusiastically.					
13	I take the initiative to solve problems in my work.					
14	I will overcome difficulties to complete the task.					
